# Calmodulin Binding to Connexin 35: Specializations to Function as an Electrical Synapse

**DOI:** 10.3390/ijms21176346

**Published:** 2020-09-01

**Authors:** Jaya Aseervatham, Xiaofan Li, Cheryl K. Mitchell, Ya-Ping Lin, Ruth Heidelberger, John O’Brien

**Affiliations:** 1 Ruiz Department of Ophthalmology & Visual Science, McGovern Medical School, The University of Texas Health Science Center at Houston, Houston, TX 77030, USA; Jaya.Aseervatham@uth.tmc.edu (J.A.); Xiaofanhouston@hotmail.com (X.L.); Cheryl.K.Mitchell@uth.tmc.edu (C.K.M.); Ya-Ping.Lin@uth.tmc.edu (Y.-P.L.); 2 Department of Neurobiology & Anatomy, McGovern Medical School, The University of Texas Health Science Center at Houston, Houston, TX 77030, USA; Ruth.Heidelberger@uth.tmc.edu; 3 The MD Anderson Cancer Center/UTHealth Graduate School of Biomedical Sciences, Houston, TX 77030, USA

**Keywords:** Cx35, Cx36, electrical synapse, gap junction, calmodulin, CaMKII, surface plasmon resonance, tracer coupling

## Abstract

Calmodulin binding is a nearly universal property of gap junction proteins, imparting a calcium-dependent uncoupling behavior that can serve in an emergency to decouple a stressed cell from its neighbors. However, gap junctions that function as electrical synapses within networks of neurons routinely encounter large fluctuations in local cytoplasmic calcium concentration; frequent uncoupling would be impractical and counterproductive. We have studied the properties and functional consequences of calmodulin binding to the electrical synapse protein Connexin 35 (Cx35 or gjd2b), homologous to mammalian Connexin 36 (Cx36 or gjd2). We find that specializations in Cx35 calmodulin binding sites make it relatively impervious to moderately high levels of cytoplasmic calcium. Calmodulin binding to a site in the C-terminus causes uncoupling when calcium reaches low micromolar concentrations, a behavior prevented by mutations that eliminate calmodulin binding. However, milder stimuli promote calcium/calmodulin-dependent protein kinase II activity that potentiates coupling without interference from calmodulin binding. A second calmodulin binding site in the end of the Cx35 cytoplasmic loop, homologous to a calmodulin binding site present in many connexins, binds calmodulin with very low affinity and stoichiometry. Together, the calmodulin binding sites cause Cx35 to uncouple only at extreme levels of intracellular calcium.

## 1. Introduction

Cell–cell communication is an essential feature of tissue organization in multicellular organisms. Nearly all tissues engage in one form of cell–cell communication via gap junctions, direct cell to cell channels that permit limited diffusion of ions and small molecules between contacting cells [1]. Gap junctions provide a means to propagate signals, either chemical or electrical, from one cell to another. However, this facility brings with it a danger in that signals emanating from a cell under severe stress could propagate that stress to its neighbors [2].

Evolution has settled on calcium as a central signaling molecule. A number of robust transport mechanisms pump calcium out of the cell and into intracellular membrane-bound compartments so that the free calcium concentration in the cytoplasm is typically in the tens of nanomolar, many orders of magnitude smaller than outside the cell. In concert, a wide variety of calcium-dependent signaling pathways have evolved that respond to nanomolar to micromolar increases in intracellular calcium [3]. This sensitive signaling also allows a cell to report its own impending doom. A simple loss of energy supply that starves the transport mechanisms will cause intracellular calcium concentration to rise well beyond its normal range. Indeed, a variety of pathways have evolved to respond to these abnormal levels of intracellular calcium with an orderly programed death of the cell [4]. This may be efficient, but if those signaling pathways are instigated in the cell’s neighbors, a chain reaction of death mediated by cell–cell communication awaits.

Not surprisingly, among the orderly mechanisms of cell death is the shutting down of cell–cell communication so as not to endanger the neighbors. It is a nearly universal property of gap junctions that high levels of intracellular calcium trigger their uncoupling [5,6]. It is also a nearly universal feature of gap junctions that the small calcium binding protein calmodulin is a component of the mechanism that responds to high intracellular calcium with disruption of cell–cell coupling [7,8]. Among the connexins, gap junction proteins found in chordates, calmodulin binding sites are very frequently found. Calmodulin binding to these sites almost exclusively leads to calcium-dependent closure of the channels [8,9], providing a general mechanism to uncouple cells in the presence of high calcium.

A caveat to the dependence on calcium signaling to decouple a stressed cell from its neighbors is the problematic implementation of such a function in excitable cells. Neurons use quite large transient increases in calcium to trigger chemical synaptic transmission, and post-synaptic responses may include substantial calcium influx. Gap junctions that function as electrical synapses in such cells may be subjected to these large changes in calcium. Do they shut down with every synaptic impulse? This would seem to be impractical, and would limit the efficacy of electrical synaptic transmission.

In this study, we have examined the properties and functional outcomes of calmodulin binding to Connexin 35 (Cx35 or gjd2b), homologous to mammalian Cx36 (or gjd2). Cx35/36 belongs to the small, evolutionarily distinct delta subgroup of the connexin family [10,11]. Its expression is almost exclusively in the nervous system [12,13,14,15], and indeed in neurons [16], with the exception of its expression in insulin-secreting pancreatic beta cells [17]. Many of its functional properties suggest that Cx35/36 is specialized to function as an electrical synapse [18,19]. Previous work has identified a conserved calmodulin binding site on these connexins [20,21], but the functional relevance of calmodulin binding has been unclear. We find that calmodulin binding uncouples Cx35 gap junctions, but that specializations in the calmodulin binding sites of Cx35 make it less sensitive to elevated intracellular calcium than many connexins. This permits the connexin and its calcium-dependent plasticity mechanisms to function unhindered during physiological excursions in calcium concentration. These characteristics paint a picture of a protein specialized to maintain its function in the dynamic and calcium-rich environment of the electrical synapse.

## 2. Results

### 2.1. Identification of Calmodulin Binding Sites in Connexin 35

**Figure 1:**
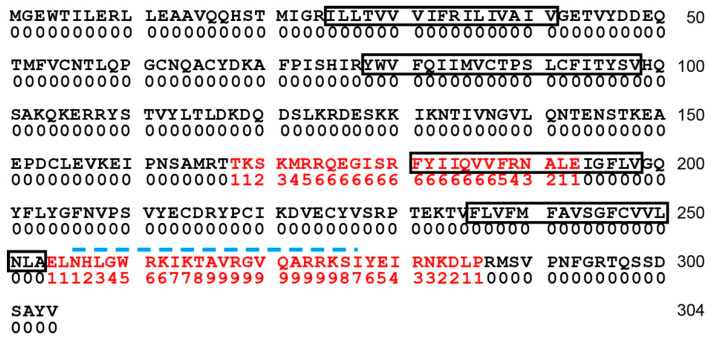
Amino acid sequence of perch Cx35 analyzed for potential calmodulin binding sites via the Calmodulin Target Database [23]. Transmembrane domains are denoted with black outlines and cumulative amino acid sequence numbers are displayed to the right of each line. Putative calmodulin binding sites are highlighted red. The binding site identified by Burr et al. [20] is denoted by the blue dashed line.

Calcium signaling is very important for the plasticity of electrical synapses composed of Cx36 and its non-mammalian orthologue Cx35 and related connexins. We previously identified a calmodulin binding site in the C-terminus of Cx36 that is conserved in all related connexins [20], and which has been studied further by Siu et al. [21]. The calmodulin binding site displayed high nanomolar to low micromolar affinity for calmodulin, very rapid calmodulin binding and dissociation kinetics, and a K_½_ for calcium around 3 micromolar [20], suggesting that it binds to calmodulin transiently when intracellular calcium is raised well above resting levels. This would be consistent with calcium-driven uncoupling of gap junctions, as has been observed for most connexins, but not with a long-term stable association between Cx36 and calmodulin, as has been implicated for one of the calmodulin binding sites in Cx32 [22]. We wished to understand better how calmodulin binding regulates behavior of Cx36-like proteins. As a first step, we used a motif search through the Calmodulin Target Database [23] to analyze the sequence of perch Cx35 for potential calmodulin binding sites. Figure 1 shows this analysis, with calmodulin binding motif scores listed below the sequence. The binding site identified by Burr et al. [20] (blue dashed line in Figure 1) scored highly in this analysis. In addition, a second potential calmodulin binding site with somewhat lower scores was identified spanning the end of the intracellular loop domain and a portion of the third transmembrane domain. This site has been previously recognized by Alev et al. [24] in mammalian Cx36. Burr et al. [20] had studied the intracellular loop empirically, finding no calmodulin binding, but the clone used for that study did not include any of the amino acid sequence within the third transmembrane domain. A different construct would be necessary to examine this site.

**Figure 2:**
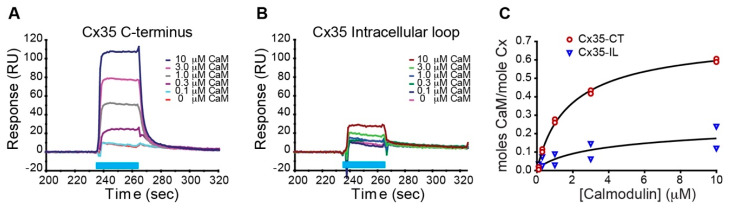
Surface plasmon resonance analysis of calmodulin binding to Cx35 C-terminal and Intracellular loop domain GST-Sumo fusion proteins. (**A**,**B**) Reference (GST-Sumo)-subtracted response traces to analyte flow over flowcells containing Cx35 C-terminus (**A**) and Cx35 Intracellular loop (**B**). Calmodulin injection at the concentrations indicated in the key is annotated by the blue bar. 1 mM free Ca^2+^ was present in the analyte solution throughout the traces. (**C**) Linear plots of calmodulin bound per mole of connexin reveal saturable calmodulin binding to both domains (*n* = 2 experiments from one chip).

To examine calmodulin binding to Cx35, we developed GST-Sumo fusion constructs of perch Cx35 intracellular loop (Cx35-IL) and C-terminal (Cx35-CT) domains. The intracellular loop construct contained amino acids 101–192, encompassing the full intracellular loop and portions of the third transmembrane domain including the potential calmodulin binding site; the C-terminal construct contained the full C-terminal domain from amino acids 251–304. We performed surface plasmon resonance (SPR) binding measurements with the Cx35 fusion proteins immobilized on the chip surface and calmodulin in analyte solution. The GST-sumo carrier protein served as a reference. Figure 2 shows a series of reference-subtracted binding responses of the C-terminus (Figure 2A) and Intracellular loop (Figure 2B) to 30-s injections of 0 to 10 µM calmodulin; 1 mM free calcium was present throughout the traces. As observed previously [20], calmodulin bound very rapidly to the C-terminal domain and dissociated rapidly in the continued presence of calcium. The intracellular loop domain also showed a weak binding response, with extremely rapid association and dissociation kinetics. Figure 2C shows binding curves for Cx35-CT and Cx35-IL derived from two experiments using the same chip. In a larger series of experiments, Cx35-CT had a first order Kd of 1.38 µM and Bmax of 0.74 moles calmodulin/mole connexin (*n* = 6), while Cx35-IL had a first order Kd of 6.29 µM and Bmax of 0.32 moles calmodulin/mole connexin (*n* = 3). Because of the relatively low stoichiometry of binding and the assessment that a portion of the putative intracellular loop binding site may be inaccessible due to its position within a transmembrane domain, we opted not to study its properties further.

### 2.2. Mutational Analysis of the Cx35 C-Terminal Calmodulin Binding Site

**Figure 3:**
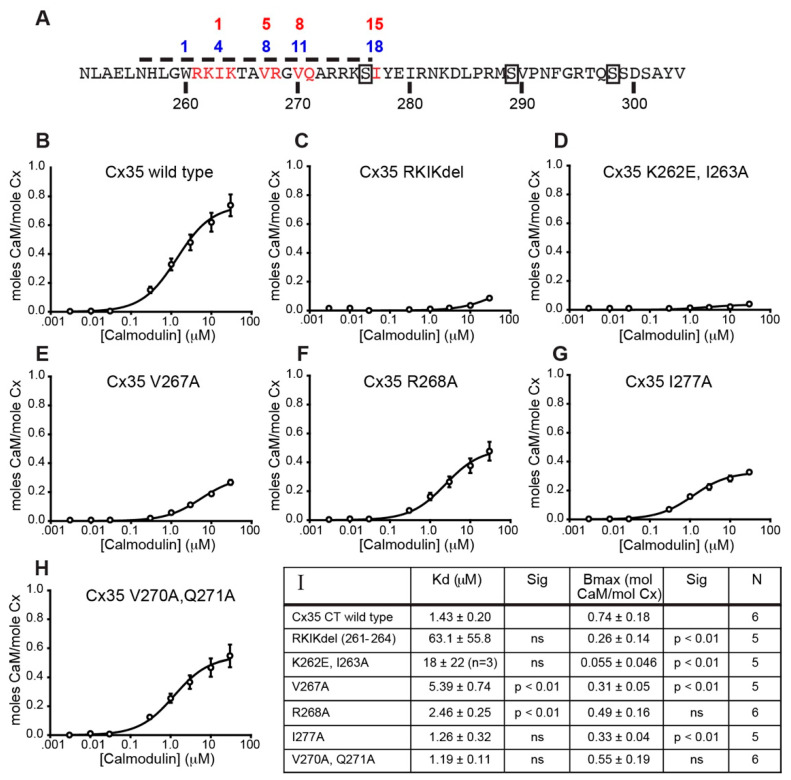
Mutational analysis of the Cx35 C-terminal calmodulin binding site. (**A**) Amino acid sequence of the perch Cx35 C-terminus. The calmodulin binding site identified by Burr et al. [20] is indicated by the dashed line. Putative hydrophobic anchor residues beginning with I263 are numbered above the sequence in red; an alternative set of anchor residues based on Siu et al. [21] is numbered above in blue. Residues highlighted red were studied by mutagenesis. Boxes indicate protein kinase phosphorylation sites that have been mapped. (**B**) Binding curve for wild type Cx35 C-terminus fit with a ligand binding model. Data are means ± SEM of 6 experiments. Best fit binding parameters are shown in (**I**). (**C**–**H**) Binding curves for Cx35 C-terminus mutants; the mutations are indicated in the title of the panel. Data are means ± SEM for 5 to 6 experiments. (**I**) Best-fit first-order binding parameters and n of experiments performed for each of the constructs shown in (**B**–**H**) Statistical comparisons to Cx35 wild type using Brown-Forsythe ANOVA with Dunnett’s multiple comparisons are also shown.

To better understand the physical properties of the C-terminal calmodulin binding site and to develop mutants that could be used to study the functional significance of calmodulin binding, we undertook a mutagenesis study of this site. Calmodulin binds to target proteins containing a variety of target motifs characterized by bulky hydrophobic residues with certain spacings. Some common calcium-dependent calmodulin binding sites have bulky hydrophobic anchor residues at positions 1 and 10 or 1, 5 and 10 (termed 1–10 motifs), or at positions 1 and 14 (1–14 motifs), with variants having additional anchors at positions 5 and/or 8; basic residues throughout the site can also be important [25,26]. Another type of target motif termed the “IQ” motif is often associated with calcium-independent binding to calmodulin [27]. Calmodulin-binding proteins often exhibit several overlapping motifs [26]. Figure 3A shows the cytoplasmic C-terminal domain of Cx35 with the previously-identified calmodulin binding site indicated by the dashed line. Inspection of the encompassed sequence revealed an arrangement of basic and hydrophobic amino acids somewhat consistent with the 1–14 class of motifs, starting with Isoleucine 263 at position 1. The hydrophobic anchor residues for this putative site are numbered above the sequence in red, with bulky hydrophobic residues present at positions 1, 5, 8 and anomalously at 15 rather than 14. Recently, Siu et al. [21] have identified Tryptophan 277 of rat Cx36, equivalent to W260 of Cx35, as an anchor residue for calmodulin binding. An alternative motif numbering using this as residue 1 is shown in blue above the sequence; using this residue as anchor residue 1 also fails to yield any canonical motifs. In addition, Cx35 has a potential IQ motif beginning at V270 and containing many of the expected subsequent basic and hydrophobic residues. This site is not conserved in mammalian Cx36, which has an alanine at position 287, equivalent to V270 of Cx35.

To study which of these residues might be involved in calmodulin binding, we made a series of mutants in the C-terminal domain fusion protein, targeting residues marked in red in Figure 3A. We studied the effects of each mutant in calmodulin binding experiments using SPR. Figure 3B shows Cx35 wild type C-terminus binding to calmodulin at concentrations ranging from 3 nM to 30 µM in 1 mM free Ca^2+^. Binding parameters, shown in Figure 3I, were Kd = 1.43 ± 0.20 µM and Bmax = 0.74 ± 0.18 moles calmodulin/mole Cx35, *n* = 6 experiments. A deletion of 4 amino acids, R261–K264 (Cx35 RKIKdel), encompassing the predicted number 1 hydrophobic anchor residue and surrounding basic residues largely abolished calmodulin binding (Figure 3C). The calculated Kd was 63 ± 56 µM (*n* = 5), which was not statistically different than wild type Cx35-CT (Brown-Forsythe ANOVA with Dunnett’s multiple comparisons, *p* = 0.256; Figure 3I) due to the very poor fits of the ligand binding model to the data and consequently very high variability. Bmax was 0.26 ± 0.14 moles calmodulin/mole Cx35 (*n* = 5), which was significantly lower than Cx35 wild type (Brown-Forsythe ANOVA with Dunnett’s multiple comparisons, *p* = 0.0043). This indicated that the basic residues, the hydrophobic residue I263, or the 3-dimensional structure of the beginning of the C-terminus were critical for calmodulin binding to Cx35.

To refine the analysis of the early anchor residues, we made point mutations of Lysine 262 to glutamate and Isoleucine 263 to alanine. These mutations remove the putative number one hydrophobic anchor residue and disrupt the basic character surrounding this residue. Figure 3D shows that these mutations virtually eliminated calmodulin binding to the Cx35 C-terminus. The Kd for calmodulin binding could be fit in only 3 of 5 experiments and was 18 ± 22 µM (*n* = 3; *p* = 0.737) and Bmax was reduced to 0.055 ± 0.046 moles calmodulin/mole Cx35 (*n* = 5; *p* = 0.0005; Figure 3I). This confirmed that the basic character and/or the hydrophobic I263 are critical for calmodulin binding.

We then examined the number 5 hydrophobic anchor residue V267, its adjacent basic residue R628, and the potentially anomalous position 15 residue I277, each with alanine mutations (Figure 3E–G). The position 5 mutation V267A significantly increased Kd for calmodulin to 5.39 ± 0.31 µM (*n* = 5; *p* = 0.0014) and decreased Bmax to 0.31 ± 0.05 moles calmodulin/mole Cx35 (*n* = 5; *p* = 0.0067). Mutation of the basic residue R268A slightly but significantly increased Kd for calmodulin to 2.46 ± 0.25 µM (*n* = 5; *p* < 0.0001), but did not change Bmax (Bmax = 0.49 ± 0.16 moles calmodulin/mole Cx35, *n* = 5; *p* = 0.151). The position 15 mutation I277A did not significantly change Kd for calmodulin (Kd = 1.26 ± 0.32 µM, *n* = 5, *p* = 0.862), but did slightly reduce Bmax to 0.33 ± 0.04 moles calmodulin/mole Cx35 (*n* = 5; *p* = 0.0077). These experiments suggest an important role for V267 in position 5 of the putative calmodulin binding site, but less critical roles for the basic residue R268 or the potential position 15 anchor residue I277.

Finally, we examined the potential role of valine 270 in position 8 of the conventional motif and potentially in position 1 of an IQ motif in a combined mutant converting both V270 and Q271 to alanine. Figure 3H shows relatively normal calmodulin binding to this mutant. These mutations did not significantly change the Kd for calmodulin (Kd = 1.19 ± 0.11 µM, *n* = 6; *p* = 0.161) or the Bmax (Bmax = 0.55 ± 0.19 moles calmodulin/mole Cx35, *n* = 6, *p* = 0.406). These results suggest that Cx35 does not use an IQ motif for calmodulin binding and that position 8 of the conventional motif is not critical for binding.

Comparing the connexin-calmodulin interactions determined structurally by Siu et al. [21] with interactions we have determined functionally it remains unclear what type of calmodulin binding motif is present in the C-terminus of Cx35/Cx36. Our interpretations agree that V267 (using Cx35 numbering) is an important hydrophobic anchor residue, and hydrophobic interactions with I263 are also seen in their data. Siu et al. detect ionic interactions with R268 and Q271, neither of which had a substantial effect on binding in our studies. Regardless of whether W260 or I263 is labeled as the number 1 hydrophobic anchor residue, the organization of hydrophobic residues involved in binding does not fit a conventional motif. Our data would suggest a variant of a basic 1-5-14 motif in which the number 14 anchor residue is in position 15. Siu et al. data suggest a variant of a 1-8-14 motif in which the number 14 anchor residue is either alanine in position 13, or is a basic residue, rather than hydrophobic. As Mruk et al. [26] describe, several overlapping motifs are often present in calmodulin binding sites, and it is likely that binding interactions do not strictly adhere to any one model.

### 2.3. Does Calmodulin Binding Uncouple Cx35 Gap Junctions?

**Figure 4:**
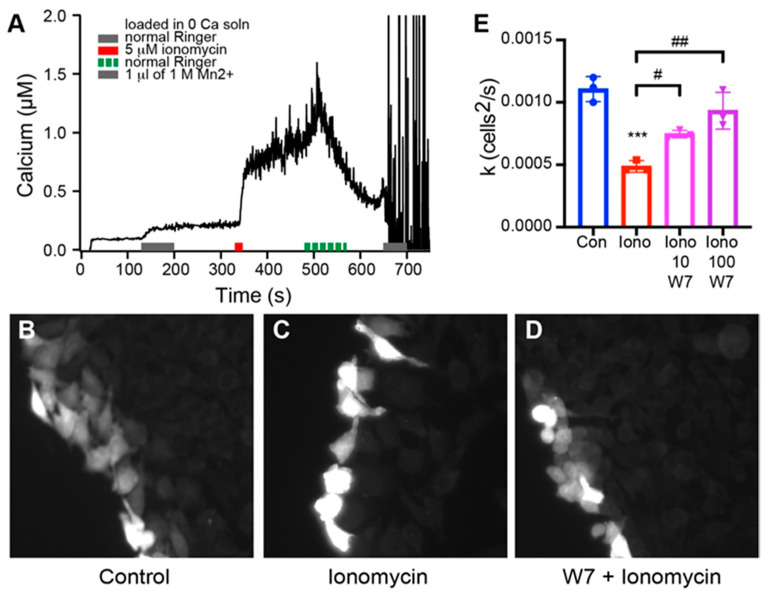
High calcium-induced uncoupling of HeLa cells expressing Cx35. (**A**) Ratiometric Ca^2+^ measurement in HeLa cells loaded with Fura-2-AM in zero Ca^2+^ Ringer solution. Gray bar indicates solution exchange into normal Ringer with 2.5 mM extracellular Ca^2+^. The red bar indicates solution exchange into normal Ringer containing 5 μM ionomycin; the green dashed bar indicates solution exchange back to normal Ringer. Direct addition of 1 μL 1 M MnCl_2_ quenches Fura-2 fluorescence as a control (resulting in the erratic ratiometric Ca^2+^ calculation seen in the trace). (**B**–**D**) Scrape-loading measurement of Neurobiotin tracer coupling in HeLa-Cx35 in normal Ringer (**B**), 5 µM ionomycin (**C**), and 5 μM ionomycin plus 100 μM W7 (**D**). (**E**) Diffusion coefficients (k) for Neurobiotin tracer coupling in HeLa-Cx35 in control, 5 μM ionomycin, and 5 μM ionomycin plus 10 μM or 100 μM W7. Bars are mean ± SD, *n* = 3 experiments, 5 measurements/experiment; *** *p* < 0.001 vs. control; # *p* < 0.05 and ## *p* < 0.01 vs. 5 μM ionomycin.

**Figure 5:**
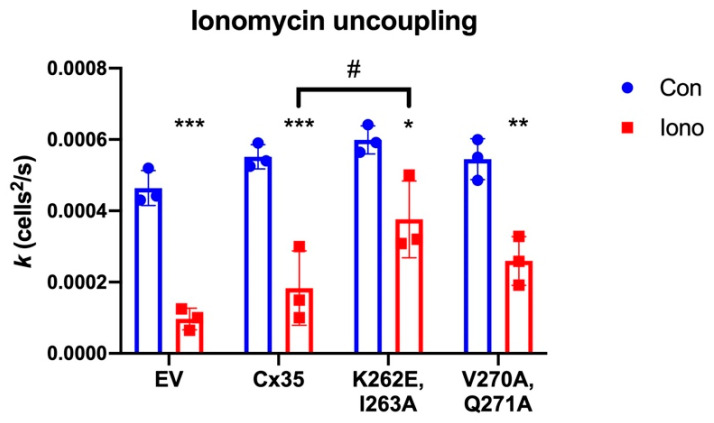
Effects of calmodulin-binding Cx35 mutants on high calcium-induced uncoupling in transiently-transfected HeLa cells. Empty vector transfection (EV) reveals the endogenous tracer coupling in HeLa cells. Ionomycin reduced this background coupling significantly. Ionomycin also reduced coupling in HeLa cells transfected with Cx35 wild type (Cx35) and the mutants K262E, I263A and V270A, Q271A. Bars are mean ± SD, *n* = 3 experiments; *** *p* < 0.001, ** *p* < 0.01 and * *p* < 0.05 vs. control; # *p* < 0.05 vs. Cx35 with 5 μM ionomycin.

The best-documented role of calmodulin in regulation of gap junction functions is its role in chemical gating to induce uncoupling of gap junctions at high intracellular calcium [8]. To investigate whether calmodulin binding is involved in Ca^2+^-induced uncoupling, we used ionomycin treatments of HeLa cells stably-transfected with Cx35. Figure 4A shows ratiometric Ca^2+^ measurement in HeLa cells loaded with Fura-2-AM in zero Ca^2+^ Ringer solution and returned to normal Ringer containing 2.5 mM extracellular Ca^2+^. Addition of 5 µM ionomycin resulted in a rapid rise in intracellular Ca^2+^, with a slow, continued rise over 3 min to approximately 1 µM free Ca^2+^ before the extracellular solution was replaced with ionomycin-free normal Ringer. This level of free Ca^2+^ is a bit below the K_1/2_ for Ca^2+^ (about 3 µM) of the C-terminal calmodulin binding site [20], but should be high enough to induce occupancy of a fraction of the C-terminal sites. Neurobiotin tracer coupling measurements of HeLa cells stably transfected with Cx35 over the course of 12 min exposure to the same levels of ionomycin and extracellular Ca^2+^ resulted in potent uncoupling of the cells (Figure 4B,C). Treatment of the cells with 100 µM calmodulin inhibitor W7 rescued coupling (Figure 4D), suggesting that the uncoupling depended on calmodulin. We measured the diffusion coefficients for Neurobiotin tracer transfer in the Cx35-transfected HeLa cells using a compartmental diffusion model [28,29]. Ionomycin significantly uncoupled HeLa cells expressing Cx35 (Figure 4E; One-way ANOVA with Sidak’s multiple comparisons, *p* = 0.0001, *n* = 3 experiments, 5 measurements per experiment). Both 10 µM and 100 µM W7 treatments significantly increased coupling above the level in ionomycin (One-way ANOVA with Sidak’s multiple comparisons: 10 µM W7, *p* = 0.0275, *n* = 3; 100 µM W7, *p* = 0.0011, *n* = 3). These experiments suggest that calmodulin binding uncouples Cx35, in contradiction to a report that calmodulin binding enhances Cx36 coupling [21]. However, our control experiments indicated that the connexin background in untransfected HeLa cells is also uncoupled by the same ionomycin treatment (not shown, but see empty vector control data in Figure 5), complicating interpretation of these experiments. Thus, while it is clear that calmodulin is responsible for high Ca^2+^-induced uncoupling of gap junctions in Cx35-transfected HeLa cells, it is not entirely clear to what extent Cx35 itself was uncoupled.

To refine our studies of the functional effects of calmodulin binding to Cx35, we employed mutations in the C-terminal calmodulin binding site that we identified in the in vitro binding studies (Figure 3). The mutation K262E, I263A essentially eliminates calmodulin binding to the C-terminal site while maintaining the spacing of residues and presumably any helical structure of the domain. The mutation V270A, Q271A has very little effect on calmodulin binding. We introduced both of these mutations into the full-length untagged Cx35 to perform tracer coupling experiments in transiently-transfected HeLa cells.

We also made efforts to eliminate the connexin background in HeLa cells in order to perform functional experiments. We used a HeLa cell line that we have recently developed in which the endogenous Cx45 alleles have been mutated by CRISPR, suppressing its expression [30]. In addition, we also transfected the cells with a construct expressing a short hairpin RNA targeted to Cx43, the other connexin expressed significantly in HeLa cells [31]. These manipulations somewhat reduce the plasma membrane trafficking of transfected Cx35, but leave functional regulation of Cx35 intact [30]. Figure 5 shows the effects of 5 µM ionomycin treatment on tracer coupling in these transiently-transfected cells. In spite of our efforts to reduce background, the cell line transfected with pcDNA empty vector (EV control) still showed some tracer coupling that was significantly reduced by ionomycin treatment (Two-way ANOVA with Tukey’s multiple comparisons, *p* < 0.0001, *n* = 3 experiments). Cells transfected with each of the Cx35 constructs supported a similar amount of tracer coupling, and each was significantly uncoupled by 5 µM ionomycin (Figure 5). However, the extent of uncoupling differed among the mutants (Two-way ANOVA with Tukey’s multiple comparisons; details follow). Coupling in Cx35 wild type-transfected cells was reduced 67% by ionomycin treatment (*p* < 0.0001, *n* = 3 experiments). In contrast, coupling was reduced only 37% in the K262E, I263A mutant that eliminates calmodulin binding to the C-terminal site (*p* = 0.016, *n* = 3 experiments), and the ionomycin-treated coupling was 105% higher than that of ionomycin-treated Cx35 wild type (*p* = 0.046, *n* = 3 experiments). In the V270A, Q271A mutant that had a very modest effect on calmodulin binding, ionomycin reduced coupling by 52% (*p* = 0.0018, *n* = 3 experiments) to a level that was not statistically different than that of Cx35 wild type (*p* = 0.852, *n* = 3 experiments). Thus, while it is likely that some of the uncoupling observed in Cx35-transfected HeLa cells is due to uncoupling of the remaining endogenous connexins, loss of calmodulin binding to Cx35 prevented a portion of the uncoupling that can be attributed to Cx35, demonstrating that calmodulin binding to the C-terminal site uncouples Cx35.

### 2.4. Does Calmodulin Binding to Cx35 Influence CaM Kinase-Mediated Potentiation?

Calmodulin-dependent protein kinase II (CaMKII) has been demonstrated to phosphorylate Cx36 [24] at a site that overlaps the calmodulin binding site (Figure 3A), potentiating its coupling [32,33]. Furthermore, CaMKII has been shown to bind directly to Cx36 through a site that overlaps with Cx36′s calmodulin binding site [24]. This arrangement suggests that there could be more subtle functions of calmodulin binding to Cx36. Perhaps calmodulin can serve as a bridge between Cx36 and the kinase, modulating the ability of CaMKII to dock with Cx36; perhaps calmodulin binding blocks phosphorylation of the S276 site, or blocks CaMKII docking entirely. As a first step to investigate these hypotheses, we examined the glutamate-driven potentiation of Cx35 coupling. We have recently shown that endogenous glutamate receptors in HeLa cells potentiate Cx36 coupling by activation of endogenous CaMKII [31]. While the exact level of free Ca^2+^ achieved in these cells is not known, Moore et al. [31] found that the responses to 100 μM glutamate stimulation of Cx36-GCaMP, which measures Ca^2+^ in the immediate vicinity of the gap junctions, were about 15% of the peak response to 5 µM ionomycin. This suggests that 100 µM glutamate stimulation should raise Ca^2+^ to very low hundreds of nanomolar. We would predict that this level of free Ca^2+^ has little ability to occupy the Cx35 C-terminal calmodulin binding site unless interactions with nearby proteins or other calmodulin binding sites alter the calcium-dependence of the interaction. We used this stimulation to examine coupling of Cx35 and its calmodulin-binding mutants.

**Figure 6:**
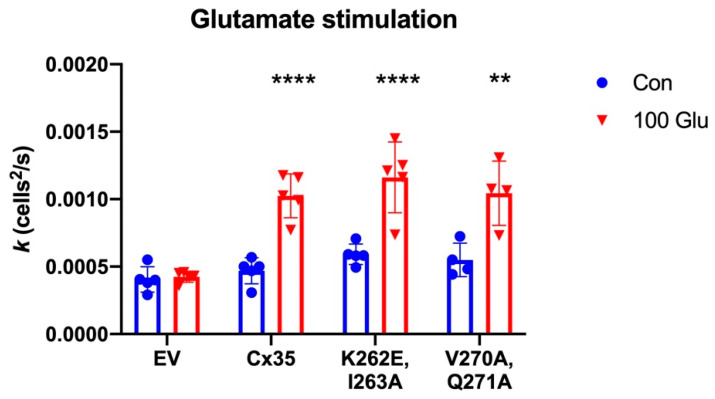
Glutamate-induced potentiation of Cx35 coupling. Diffusion coefficients (k) for Neurobiotin tracer coupling in control Ringer (Con) or Ringer plus 100 µM glutamate (100 Glu) for 20 min. Tracer coupling was not changed in empty vector-transfected HeLa cells (EV) by addition of glutamate. Tracer coupling was significantly increased in wild type Cx35 and the calmodulin binding site mutants K262E, I263A and V270A, Q271A. Bars are mean ± SD; *n* = 4 to 5 experiments; **** *p* < 0.0001, ** *p* < 0.01 vs. control.

Figure 6 shows that 100 µM glutamate stimulation for a total of 20 min did not change background coupling in HeLa cells transfected with empty pcDNA vector (EV; Two-way ANOVA with Tukey’s multiple comparisons, *p* = 0.999, *n* = 5 experiments), while coupling in wild type Cx35-transfected cells increased 118% (*p* < 0.0001, *n* = 5). In the K262E, I263A mutant that prevented calmodulin binding, glutamate increased tracer coupling by 97% (*p* < 0.0001, *n* = 5). Neither the control level of coupling nor the glutamate-stimulated level of coupling differed in the K262E, I263A mutant from Cx35 wild type (control: *p* = 0.906, *n* = 5; 100 glutamate: *p* = 0.842, *n* = 5). In the V270A, Q271A mutant that had little effect on calmodulin binding, glutamate stimulation increased tracer coupling by 90% (*p* = 0.0017, *n* = 4), and again neither control nor glutamate-stimulated coupling differed from wild type Cx35 (control: *p* = 0.993, *n* = 5 Cx35 WT, *n* = 4 V270A, Q271A; 100 glutamate: *p* = 0.999, *n* = 5 Cx35 WT, *n* = 4 V270A, Q271A). These results suggest that calmodulin binding to the C-terminal calmodulin binding site does not contribute to CaMKII-mediated potentiation of coupling during glutamate stimulation.

### 2.5. Do Calmodulin Binding Sites Affect Protein Trafficking?

**Figure 7:**
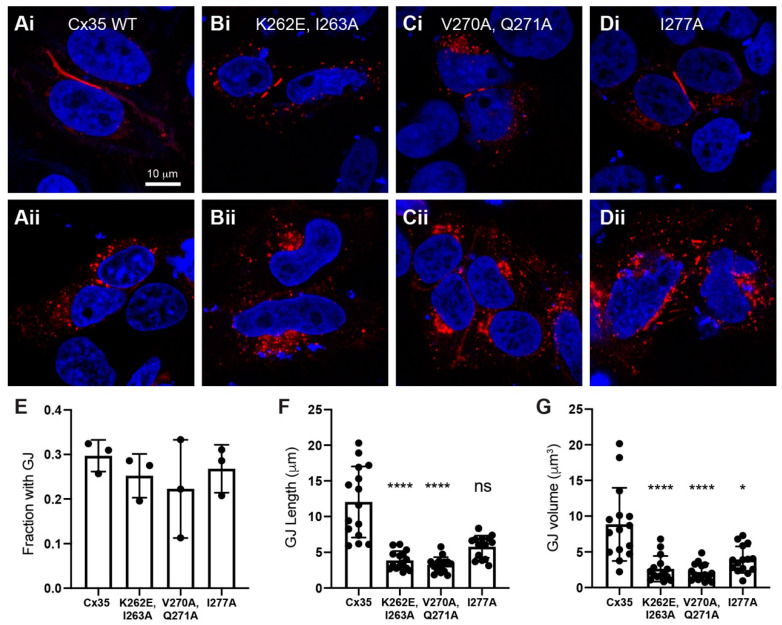
Trafficking and gap junction formation of Cx35 calmodulin binding site mutants. (**A**–**D**) Immunofluorescence labeling of Cx35 (red), with DAPI-labeled nuclei (blue). Two examples each of cells transiently transfected with wild type Cx35 (**A**), K262E, I263A mutant that exhibits no calmodulin binding (**B**), V270A, Q271A mutant that exhibits nearly normal calmodulin binding (**C**), and I277A mutant that exhibits mildly reduced calmodulin binding (**D**). Scale bar in Ai applies to (**A**–**D**). (**E**) Fraction of Cx35-expressing cell pairs with visible gap junctions; *n* = 3 experiments. (**F**) Quantitation of gap junction length, and **G** gap junction volume; *n* = 15 gap junctions from 3 experiments; Kruskal–Wallis test: **** *p* < 0.0001, * *p* < 0.05, ns = not significant vs. Cx35 wild type. Bars in (**E**–**G**) are mean ± SD.

A calmodulin binding site in the C-terminus of Cx32 has been demonstrated to be important for the proper oligomerization of Cx32 monomers into connexons [34] and calmodulin has been proposed to enter an association with Cx32 before gap junctions are formed [22]. Such an early association of calmodulin during the assembly and trafficking of connexins is consistent with the observation that Cx36 associates with calmodulin in intracellular vesicles [21]. In order to understand whether the Cx35 C-terminal calmodulin binding site affected expression or trafficking of Cx35, we examined Cx35 calmodulin binding site mutants by immunofluorescence microscopy. Because the loss of Cx45 was found to compromise forward trafficking of Cx35 in HeLa cells [30], we performed these studies in normal HeLa cells. In transiently-transfected HeLa cells, wild type Cx35 displayed a wide range of gap junction sizes (Figure 7A,F,G) with a variable fraction of protein retained in intracellular vesicles (e.g., Figure 7Ai vs. Aii). We examined three of the C-terminal calmodulin binding site mutants in these transfection experiments: Cx35 K262E, I263A, which essentially eliminates calmodulin binding (Figure 3D), Cx35 V270A, Q270A, which has minimal effects on calmodulin binding (Figure 3H), and Cx35 I277A, which modestly reduces calmodulin binding (Figure 3G). All of the calmodulin binding site mutants empirically displayed greater retention of protein in intracellular vesicles and the presence of only rather small gap junctions (Figure 7B–D). Assessment of the fraction of contacting cell pairs expressing Cx35 with visible gap junctions (Figure 7E) revealed no difference in the ability of any of the mutants to form gap junctions (one-way ANOVA with Dunnett’s multiple comparisons, *p* = 0.626, *n* = 3 experiments).

Because gap junctions made by the calmodulin binding site mutants appeared to be smaller than wild type, we measured length and volume of 15 arbitrarily-selected gap junctions of each type from three separate experiments. Figure 7F shows that the length of gap junctions was significantly smaller in Cx35 mutants K262E, I263A and V270A, Q270A than wild type Cx35 (Kruskal-Wallis test with Dunn’s multiple comparisons, *n* = 15: Cx35 mean rank 51.10, K262E, I263A mean rank 20.13, *p* < 0.0001; Cx35 mean rank 51.10, V270A, Q270A mean rank 14.70, *p* < 0.0001). While its mean length was smaller, Cx35 mutant I277A did not reach statistical significance in a non-parametric test (Kruskal-Wallis test with Dunn’s multiple comparisons, *n* = 15: Cx35 mean rank 51.10, I277A mean rank 36.07, *p* = 0.055). Mean gap junction volume (Figure 7G) was significantly smaller in all of the mutants (*n* = 15 for all comparisons: Cx35 mean rank 48.93, K262E, I263A mean rank 21.43, *p* < 0.0001; Cx35 mean rank 48.93, V270A, Q270A mean rank 18.33, *p* < 0.0001; Cx35 mean rank 48.93, I277A mean rank 33.30, *p* = 0.0427). Thus, disruption of the Cx35 C-terminal calmodulin binding site reduced the average size of Cx35 gap junctions regardless of the effect of the mutation on calmodulin binding.

## 3. Discussion

Calmodulin binding has emerged to be a nearly universal characteristic of gap junction proteins [8]. The members of the delta family of connexins are no exceptions to this rule, with a calmodulin binding site known in the C-terminal cytoplasmic domain of these connexins [20,21]. The delta family of connexins is unusual among connexins in that its members are expressed primarily in neurons, where they form electrical synapses. This places them in an environment in which fluctuations in the level intracellular calcium occur frequently. Indeed, for electrical synapses located close to chemical synapses, these fluctuations are likely to be very large and free Ca^2+^ should routinely rise transiently to a level that drives some occupancy of the C-terminal binding site by calmodulin. Thus, the role of calmodulin binding in functional regulation of the electrical synapses is potentially of some importance.

### 3.1. Calmodulin Binding Uncouples Cx35 Gap Junctions

Our experiments in this study showed that ionomycin treatment in HeLa cells caused uncoupling of Cx35 gap junctions, an effect suppressed in the K262E, I263A mutant that essentially eliminates calmodulin binding to the C-terminal site. The experiments also showed residual uncoupling in cells transfected with the K262E, I263A mutant. We believe this to be the response of the remaining endogenous connexins in the cells. The treatment we used increased intracellular free Ca^2+^ up to approximately 1 µM, a level below Cx35′s K_1/2_ for calcium and suitable to drive about 1/3 occupancy of the C-terminal calmodulin binding site [20] if the calcium dependence of binding in vivo mirrors that of in vitro experiments. If this is indeed the case, it suggests that calmodulin binding to only a fraction of connexins within a connexon is adequate to uncouple the gap junction.

Our finding that calmodulin binding to perch Cx35 uncouples the gap junction contradicts the findings of Siu et al. [21], who found that calmodulin binding increased coupling in rat Cx36-transfected Neuro2a cells. While it is possible that species differences account for this contradiction, this is unlikely. The amino acid sequences of the two proteins are 82% identical and sequence conservation in the Cx35 gene homologues is among the highest of any in the connexin gene family [13,35,36]. Furthermore, the calmodulin binding sites have very similar properties [20], and functional properties and regulation of fish and mammalian Cx35/Cx36 have proven to be essentially the same [13,29,31,37,38,39]. It is possible that other factors led to the difference in results, such as the use of Neuro2a cells and a slightly lower concentration of ionomycin in the Siu et al. study. Indeed, our preliminary experiments in which ionomycin incubation was started 10 min prior to scraping to initiate tracer loading and transfer (vs. 2 min as shown in the final experiments in Figure 5) resulted in a profound increase in tracer diffusion in cells expressing Cx35 or mutants and little to no uncoupling of endogenous connexins (Supplemental Figure S1; *n* = 1 experiment with 4–8 measurements per condition). We interpret this finding to indicate that, during the 10-min pre-incubation plus 10 min of post-scrape diffusion, the active calcium extrusion mechanisms in HeLa cells (e.g., see rapid recovery in Figure 4A) may have reduced cytoplasmic free calcium to a level below that which activates the direct calmodulin binding to Cx35, but high enough to activate CaMKII and potentiate coupling. Thus, the cell- and time-specific experimental conditions may have influenced the outcome of experiments. In our case, the Neurobiotin tracer transfer technique has proven to be quantitative and reliable [29,40] but lacks time resolution, necessitating the carefully-timed and somewhat heavy-handed approach to assess the true effects of high intracellular calcium. We believe that the experiments we present make a strong case that calmodulin binding to the C-terminal site of Cx35 uncouples the gap junctions.

**Figure 8:**
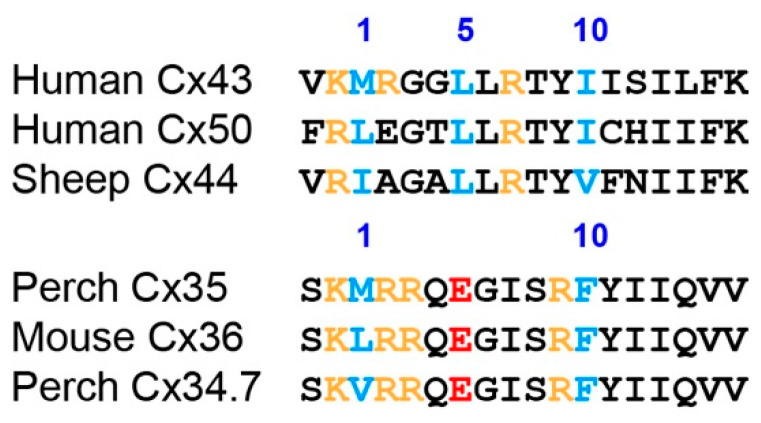
Sequences of cytoplasmic loop juxtamembrane calmodulin binding sites for human Cx43, Cx50, sheep Cx44 [9], Perch Cx35, Mouse Cx36 and Perch Cx34.7. The hydrophobic anchor residues making up the 1, 5, 10 motifs are designated above the sequences and highlighted in blue. Basic residues within the motif are highlighted orange. The position 5 anchor residue in Cx35/36 and Cx34.7 is replaced with an acidic glutamate (highlighted red), disrupting the motif.

Curiously, while our experiments employed 5 µM ionomycin with 2.5 mM extracellular Ca^2+^ and required careful timing to capture the calmodulin-induced uncoupling of Cx35, a number of other connexins are uncoupled by milder conditions. For example, connexins 43, 44 and 50 have all been shown to be uncoupled in either HeLa or Neuro2a cells with a treatment of 1 µM ionomycin with 1.8 mM extracellular Ca^2+^ [41,42,43,44], conditions very similar to those used by Siu et al. [21]. Indeed, calcium measurements in Cx43-transfected Neuro2a cells indicated that this treatment reached low hundreds of nanomolar free Ca^2+^, achieving potent and sustained uncoupling of the gap junctions [43]. Similar levels of Ca^2+^ uncouple Cx43 in HeLa cells [45]. Thus, Cx35 uncoupling is substantially less sensitive to intracellular free Ca^2+^ than many connexins. The primary calmodulin binding site responsible for uncoupling these connexins is a juxtamembrane site at the end of the cytoplasmic loop employing a basic 1, 5, 10 motif [9] (Figure 8). Indeed, such a site is predicted to be present in a large number of other connexins [8]. Figure 8 shows that this site is homologous to the cytoplasmic loop site predicted in Cx35 and Cx36, and a homologous site in Cx34.7. We previously found no calmodulin binding to this site when only residues outside of the transmembrane domain were included [20], and quite low affinity and low stoichiometry of binding when a substantial portion of transmembrane domain 3, including residues potentially contributing to a calmodulin binding site, were included (Figure 2 of this study). Inspection of the sequence of the cytoplasmic loop site of Cx35 and its relatives reveals that the number 5 hydrophobic anchor residue in this motif has been replaced by an acidic glutamate, disrupting the site (Figure 8). While empirically, this site still binds calmodulin weakly (Figure 2), it clearly no longer functions effectively in vivo at high nanomolar concentrations of intracellular Ca^2+^ to promote uncoupling. The glutamate residue in this position is completely conserved in the Cx35 and Cx34.7 gene homologues in a large number of species (authors’ unpublished observations). This may be one aspect of specialization to function in the environment of the electrical synapse, and particularly in mixed synapses [46,47], where dynamic and high intracellular calcium levels are the norm and junctional coupling can be and is maintained during neural activity.

### 3.2. Competition between Calmodulin Uncoupling and CaMKII Potentiation

Another aspect of functioning in the high-calcium environment of the electrical synapse is the importance of calcium/calmodulin-dependent protein kinase function. CaMKII is critical for regulating functional plasticity of Cx36 gap junctions [24,32,33,48], increasing coupling by phosphorylating Cx36. Alev et al. [24] have shown that CaMKII binds directly to Cx36 through interactions with sites in the cytoplasmic loop and C-terminal domains that directly overlap with the calmodulin binding sites. The C-terminal site also contains a CaMKII phosphorylation site that is critical for regulating coupling [24,32]. One of the most intriguing potential ramifications of the overlap of CaMKII and calmodulin binding sites is that calmodulin binding might regulate the CaMKII interaction and thus modulate functional plasticity. We tested this hypothesis directly and found that loss of calmodulin binding to the C-terminal site had no effect on CaMKII-induced potentiation of coupling (Figure 6). Calmodulin binding to CaMKII appears to have somewhat similar calcium dependence in vitro as its binding to Cx36, requiring low micromolar concentrations to achieve half saturation [49]. However, CaMKII activation and autophosphorylation traps calmodulin [50] permitting transient interactions that activate CaMKII to be integrated to persistently activate the kinase, a key element of long-term potentiation [51]. So, while transient influx of calcium may trigger calmodulin binding to both Cx36 and CaMKII, this interaction may disappear from Cx36 when free Ca^2+^ falls below micromolar levels. In such instances, the potentiating effect of CaMKII phosphorylation will dominate the outcome. This can be very important for synapses in which activity-dependent plasticity is a central element of functional regulation [19,52,53].

### 3.3. Does Calmodulin Binding Play A Role in Cx35 Trafficking?

The final aspect of calmodulin binding to Cx35 that we studied was the effect of calmodulin binding on trafficking and gap junction formation. Siu et al. found that calmodulin association with Cx36 increased in intracellular vesicles with calcium influx, and that mutation of W277 (equivalent to W260 of Cx35) reduced the incidence of gap junctions, but not their size [21]. While we did not examine W260, mutations that we made within the calmodulin binding site did not affect the incidence of gap junction formation between pairs of cells expressing Cx35, but consistently reduced the average size of gap junctions. The latter reduction in size of gap junction plaques was not dependent on the effect of the mutation of calmodulin binding: even V270A, Q271A, which did not reduce calmodulin binding, caused a reduction of gap junction size equivalent to that caused by the K261E, I262A mutant that essentially eliminated calmodulin binding (Figure 7). This suggests that loss of calmodulin binding was not a factor contributing to reduction in gap junction size.

Brown et al. [54] have recently discovered that tubulin binds to Cx36 via a site that completely overlaps with the C-terminal calmodulin binding site. Indeed, hydrophobic and basic residues that contribute to calmodulin binding are also the determinants of tubulin binding [54]. Curiously, Brown et al. found that point mutation of every residue from K279 to G286 (equivalent to K262 to G269 of Cx35) caused a significant reduction in gap junction size, very much like the outcome of our experiments in the region of K262 to I277. This was the case despite the fact that only mutation of select residues, K281, R285 and G286 (K264, R268 and G269 of Cx35), reduced intracellular vesicle movement that was presumably dependent on tubulin [53]. Thus, the tubulin binding function of this region of Cx35 may have a significant role in the limitation of gap junction plaque size. However, it appears that still other factors are involved, implying that the early part of the C-terminus is extremely sensitive to perturbations.

### 3.4. A Nexus for Functional Regulation

It is quite striking how many functionally important interactions converge on the small portion of the beginning of the C-terminus of Cx35, its orthologues and related connexin isoforms. These interactions include calmodulin binding [20,21], tubulin binding [54], and regulatory phosphorylation by protein kinase A [29,55], protein kinase G [56] and CaMKII [24,32]. The location of this site close to the end of transmembrane domain 4 is likely not a coincidence. While the numerous basic and hydrophobic amino acids in this site are important for the interactions with calmodulin and tubulin, and are components of protein kinase target recognition motifs, they also closely resemble juxtamembrane polybasic domains that interact directly with the inner leaflets of plasma membrane phospholipid bilayers. These domains are involved in numerous interactions that regulate a wide variety of functions [57]. Furthermore, locally high concentrations of Ca^2+^ can cause these domains to dissociate from the membrane, further contributing to regulating protein functions [58].

The cytoplasmic end of the connexin 4th transmembrane domain is positioned on the outer rim of the connexon cytoplasmic face in the Connexin 26 crystal structure [59]. Here the proximal end of the C-terminal domain has access to the surrounding lipid membrane and is accessible to cytoplasmic proteins that might regulate function. A cork model of connexin gating, in which calmodulin bound to the connexin enters and occludes the channel vestibule, has been proposed for calmodulin binding to connexins [9,60,61]. This model makes sense for calmodulin binding to the N-terminus, located in the channel vestibule [59], and perhaps for sites at the end of the cytoplasmic loop that occupy the rim of the vestibule. For this to work at the C-terminal site would require a large displacement of the domain, although cross-site bridging models [62,63] would facilitate this sort of conformational change. However, it must be considered that relatively small changes, such as phosphorylation within the domain, adding negative charges, potently increases coupling through an unknown physical mechanism. It seems likely that this change promotes a change in the position of transmembrane domain 4 that enhances coupling. Likewise, calmodulin binding may promote an opposing change that suppresses coupling. These hypotheses will require further structural investigation to test.

### 3.5. Limitations of This Study

This study correlates in vitro calmodulin binding data to in vivo functional data. While the fusion proteins containing the full cytoplasmic intracellular loop and C-terminal domains are more likely to reflect natural structural properties of connexin cytoplasmic domains than do small synthetic peptides often used for such studies, they still lack the context within an intact protein with 4 transmembrane domains. It is possible that calmodulin binding to Cx35 is influenced by the structural organization of the connexin and the surrounding plasma membrane in ways not captured in the in vitro studies, or that interactions involving both binding sites may change the stability or calcium dependence of the binding interactions. We believe that the experiments with intact Cx35 with mutations that eliminate C-terminal calmodulin binding provide significant insight into the true functional importance of calmodulin binding. Further studies closely examining the relationship of intracellular calcium concentration to coupling will provide additional insight.

## 4. Materials and Methods

### 4.1. Clones

Connexin 35 cytoplasmic domains were expressed as fusion proteins in *Escherichia coli* strain BL21(DE3). In order to minimize degradation of fusion proteins in bacteria and enhance proper folding, we initially cloned cytoplasmic fragments of Cx35 as fusion proteins with human Sumo3. The full perch Cx35 C-terminus containing amino acids 251–304 and the full cytoplasmic loop plus a portion of transmembrane domain 3, consisting of amino acids 101 to 192 were amplified by PCR using Pfu polymerase (Agilent, Santa Clara, CA, USA) from PCx35-pcDNA [29] (Addgene plasmid 42920) and cloned into BsaI and XhoI sites of pE-SUMO3 (Life Sensors, Malvern, PA, USA). Enzymes used for cloning were obtained from New England Biolabs (Ipswich, MA, USA) unless otherwise noted. Mutations were introduced into the C-terminal fusion protein construct using the QuickChange site-directed mutagenesis kit (Agilent). All clones were confirmed by sequencing on both strands.

The His-tagged Sumo fusion proteins proved difficult to purify and capture for further experiments, so each Sumo-Cx35 domain construct was excised in its entirety with NcoI and XhoI and cloned into pET42c (Novagen, Madison, WI, USA) in frame with Glutathione S-transferase. The resulting GST-Sumo fusion proteins were used for subsequent binding experiments. A control clone was generated by transferring Sumo3 from the parent vector into pET42c in the same way.

The full-length perch Cx35 cDNA (PCx35-pcDNA) [29] was used for expression studies. Mutations were introduced in the full-length clone using the QuickChange site-directed mutagenesis kit (Agilent). All clones were confirmed by sequencing on both strands.

### 4.2. Surface Plasmon Resonance Studies

GST-Sumo fusion proteins of the Cx35 cytoplasmic domains and the control GST-Sumo clone were purified from *E. coli* using glutathione sepharose 4B (Amersham, Piscataway, NJ, USA) according to the manufacturer’s protocols. Surface Plasmon Resonance (SPR) studies were performed on a Bioacore 2000 (Biacore AB, Uppsala, Sweden) 4-channel instrument. Anti-GST antibodies (27-4577-01, Amersham; RRID:AB_771432) were immobilized on CM5 SPR chips (Biacore) by amine coupling using 1-ethyl-3-(3-dimethylaminopropyl)carbodiimide and *N*-hydroxysuccinimide. The GST-Sumo fusion proteins were then captured in each flowcell as previously described [20]. The control GST-Sumo protein was loaded in the first flowcell and served as a non-specific binding reference that was subtracted from all runs.

Purified calmodulin [64] was a generous gift of Neal Waxham (University of Texas Health Science Center at Houston). Calmodulin was injected in the flow buffer at concentrations ranging from 3 nM to 30 µM at a flow rate of 20 L/min. The flow buffer contained 1 mM free Ca^2+^ and 0.5 mM free Mg^2+^ with the following components: 140 mM KCl, 10 mM MOPS, pH 7.2, 5 mM HEDTA, 5 mM NTA, 9.54 mM CaCl_2_, 0.906 mM MgCl_2_, 0.05% Tween 20 [20]. A calcium-free flow buffer with 0.5 mM free Mg^2+^ was used between runs to strip any bound calmodulin off the chip; this solution contained 140 mM KCl, 10 mM MOPS, pH 7.2, 5 mM HEDTA, 5 mM EGTA, 4.62 mM MgCl_2_, 0.05% Tween 20 [20].

Data were analyzed with BIAEvaluation software (Biacore) and further processed in Microsoft Excel (Microsoft, Redmond, WA, USA). Binding data were fit to first and second order ligand binding models with Simfit (https://www.simfit.org.uk). The first order binding constants are reported, as additional terms gave only small improvements of the fits.

### 4.3. Tracer Coupling Studies

HeLa cells (catalog #CCL2, ATCC, Manassas, VA; RRID:CVCL_0030) were used between passages 7 and 20 relative to the original cell line obtained from ATCC. A derivative of this cell line in which 5 endogenous alleles of Cx45 (*GJC1*) were mutated with CRISPR [30], termed HeLa A1, was used for tracer coupling in transient transfection experiments. Cells were plated onto tissue culture coated coverslips (ThermoFisher, Waltham, MA, USA) and grown to 70–80% confluence in minimal essential medium (MEM) with essential amino acids, 10% fetal bovine serum and penicillin/streptomycin/fungizone (cell culture reagents were all from Gibco/ThermoFisher). Cells were transiently transfected with Cx35 clones in pcDNA vectors along with clone expressing an shRNA targeted to human Cx43 (*GJA1*; Cat. # TRCN0000059773, Sigma-Aldrich, St. Louis, MO, USA) using GenePorter 2 transfection reagent (Genlantis, San Diego, CA, USA) and cultured for 48 h. Prior to use in experiments, cells were rinsed twice with MEM.

A stably-transfected HeLa cell line expressing perch Cx35 from the PCx35-pcDNA vector described above has been previously described [29]. These cells were cultured and plated as described above, but were not transfected with the Cx43 shRNA construct. Cells were used in experiments 48 h after plating on coverslips, and were rinsed twice with MEM prior to experiments.

Tracer coupling was performed as previously described [29,31]. Briefly, HeLa cells were rinsed in modified Ringer solution containing 150 mM NaCl, 6.2 mM KCl, 1.2mM NaH_2_PO_4_, 1.2 mM MgSO_4_, 2.5 mM CaCl_2_, 10 mM glucose, 10 mM HEPES pH 7.4. Cells were incubated in the same medium with 100 µM glutamate + 1 mM glycine, or with control medium for 10 min at 37 °C. Fresh solutions of the same composition plus 0.1% Neurobiotin (Vector Laboratories, Burlingame, CA, USA) were added to each dish and the cells scraped with a 26-gauge needle. Scraped cells were incubated a further 10 min to allow for loading and tracer diffusion, rinsed and fixed with 4% formaldehyde in 0.1 M phosphate buffer. For experiments examining calcium-dependent uncoupling, cells were incubated with 10 µM or 100 µM calmodulin inhibitor W7 (Axxora, San Diego, CA, USA), or control medium containing vehicle (0.1% DMSO) for 8 min. The solutions were exchanged for solutions of the same composition plus 0.1% Neurobiotin and 5 µM ionomycin (Sigma, St. Louis, MO, USA), incubated for 2 min, and then scraped, allowed to incubate for 10 min of diffusion, rinsed and fixed as before. Neurobiotin in fixed cells was visualized with Cy3-strepavidin (1:250; Jackson ImmunoResearch, West Grove, PA, USA).

Labeled coverslips were imaged at 40× magnification with a digital camera using HCImage software (Hamamatsu Photonics, Bridgewater, NJ, USA) and intensity data captured in 2 µm circular regions of interest centered within each cell of a labeled cluster. Intensity and position data were fit to a linear compartmental diffusion model [28,40] using Matlab software (Mathworks, Natick, MA, USA) to calculate diffusion coefficients for Neurobiotin tracer transfer. In each experiment, 4–8 independent measurements were made on each scrape-loaded coverslip. These were averaged to generate an overall experimental average. Data points shown are the experimental averages; 3 to 5 experiments were performed for each analysis.

### 4.4. Ratiometric Calcium Measurements

HeLa cells stably-transfected with perch Cx35 were grown and plated as described above. Cells were loaded with 5 µM Fura-2, AM (Molecular Probes/ThermoFisher) in Ca^2+^-free Ringer solution (the solution above minus CaCl_2_ and with 5 mM additional NaCl to normalize solution osmolality) for 45 min in the dark at 32 °C. Ratiometric measurements were made from single loaded cells using a photodiode with an adjustable aperture to limit the emission field using a Zeiss (Thornwood, NY, USA) Axiovert 100 microscope with a 100×/1.25 NA water immersion objective. Alternating excitation at 360 nm and 388 nm was provided by a computer-controlled monochromator-based system [65,66]. Intracellular Ca^2+^ concentration was calculated from the ratio of emitted fluorescence at the two wavelengths [67] using calibration constants determined using highly-buffered solutions of known Ca^2+^ concentration [68].

### 4.5. Immunofluorescence Imaging

HeLa cells were transiently transfected with perch Cx35 or its calmodulin binding site mutants as described above and cultured for 48 h. The cells were fed with fresh medium and two hours later were fixed with 4% formaldehyde in 0.1 M phosphate buffer, pH 7.5 for 30 min, washed with 0.1 M phosphate buffer, and blocked 1 h in 10% normal donkey serum in 0.1 M phosphate buffer, 0.3% Triton X100. Cells were then immunolabeled using anti-Cx35/36 (clone 9D7.2, MAB 3043; Millipore-Sigma, Burlington, MA; RRID:AB_94636), 1:500 in phosphate-buffered saline + 0.3% Triton X100, 0.1% sodium azide (PBSTA). Washed coverslips were probed with Cy3-donkey anti-mouse secondary antibodies (1:500; Jackson ImmunoResearch) and mounted with Vectashield with DAPI (Vector Laboratories). Coverslips were imaged with a Zeiss LSM800 confocal microscope with 63×/1.4 NA objective.

### 4.6. Statistical Analyses

Statistical analyses were performed in Prism software (GraphPad Software, San Diego, CA, USA). Calmodulin binding parameters from 5 to 6 experiments were analyzed with One-way ANOVA. In the case of Cx35 RKIKdel and Cx35 K262E, I263A mutants, Kd was fit very poorly and varied over a wide range; in 2 of 5 experiments, no Kd could be fit to Cx35 K262E, I263A binding data. For this reason, we adopted a Brown-Forsythe ANOVA to account for differences in standard deviation of the binding parameters of these mutants compared to wild type and other mutants; Dunnett’s multiple comparison tests were used.

Tracer coupling data were analyzed with ordinary One-way ANOVA with Sidak’s multiple comparisons (for experiments comparing drug treatments of wild-type Cx35) or ordinary Two-way ANOVA with Tukey’s multiple comparisons (for comparisons of ionomycin or glutamate responses of wild type Cx35 and Cx35 mutants).

Analyses of the frequency of gap junctions between pairs of expressing cells employed One-way ANOVA with Dunnett’s multiple comparisons. Analyses of gap junction size parameters employed non-parametric tests because the distribution of sizes were skewed, depending on the abundance of very large plaques. A Kruskal–Wallis test with Dunn’s multiple comparisons was used.

## Supplementary Materials

Supplementary materials can be found at https://www.mdpi.com/1422-0067/21/17/6346/s1. Figure S1. Tracer coupling in HeLa cells transiently transfected with Cx35 and its mutants with 10 min pre-incubation with 5 µM ionomycin.

## Abbreviations

MDPI	Multidisciplinary Digital Publishing Institute
DOAJ	Directory of open access journals
CaMKII	Calmodulin-dependent protein kinase II
Cx35	Connexin 35 (gjd2b)
Cx36	Connexin 36 (gjd2)
EGTA	Ethylene glycol-bis(2-aminoethylether)-N,N,N′,N′-tetraacetic acid
GST	Glutathione S-transferase
HEDTA	N-(2-Hydroxyethyl)ethylenediamine-N,N′,N′-triacetic acid
HEPES	N-(2-Hydroxyethyl)piperazine-N′-(2-ethanesulfonic acid)
MEM	Minimal essential medium
MOPS	3-(N-morpholino)propanesulfonic acid
NTA	Nitrilotriacetic acid
W7	N-(6-Aminohexyl)-1-naphthalenesulfonamide hydrochloride
