# Supplementary material for: Calmodulin Binding to Connexin 35: Specializations to Function as an Electrical Synapse
Source: Int J Mol Sci. 2020 Sep 1;21(17):6346. doi: 10.3390/ijms21176346 (PMC7504508; doi:10.3390/ijms21176346)
Supplement: Supplementary file 1 [file ijms-21-06346-s001.docx]

|  |
| --- |
| **Supplemental Figure S1**. Tracer coupling in HeLa cells transiently transfected with empty pcDNA vector (EV), wild type Cx35, calmodulin binding-deficient Cx35 mutant K262E, I263A, and Cx35 mutant V270A, Q271A with largely normal calmodulin binding. Bars show mean ± SD for 4-8 independent measurements of diffusion coefficient for Neurobiotin tracer transfer from a single experiment; individual measurements are shown. Each treatment includes 10 minutes pre-treatment with the compounds indicated plus 10 minutes with the same compounds following scraping to load the cells with Neurobiotin. Treatments are 0.05% DMSO in Ringer solution (Con), 5 μM ionomycin (Iono – 10 pre), 5 μM ionomycin + 100 μM W7 (100 W7), and 100 μM Glutamate + 1 mM Glycine (Glu). Statistical comparisons are two-way ANOVA with Tukey’s multiple comparisons: ****p < 0.0001 and **p < 0.01 vs. control treatment of the same construct; ###p < 0.001 and ##p < 0.01 vs. ionomycin treatment of the same construct. |
